# Editorial: Deep brain stimulation think tank: updates in neurotechnology and neuromodulation, volume IV

**DOI:** 10.3389/fnhum.2024.1441212

**Published:** 2024-06-27

**Authors:** Kara A. Johnson, James J. Giordano, Adolfo Ramirez-Zamora, Marta San Luciano, Svjetlana Miocinovic, Michael S. Okun, Joshua K. Wong

**Affiliations:** ^1^Norman Fixel Institute for Neurological Diseases, University of Florida, Gainesville, FL, United States; ^2^Department of Neurology, University of Florida, Gainesville, FL, United States; ^3^Departments of Neurology and Biochemistry, and Neuroethics Program, Georgetown University Medical Center, Washington, DC, United States; ^4^Department of Neurology, University of California, San Francisco, San Francisco, CA, United States; ^5^Department of Neurology, Emory University, Atlanta, GA, United States

**Keywords:** deep brain stimulation, neuroimaging, neurophysiology, movement disorders, interventional psychiatry

Deep brain stimulation (DBS) is a rapidly advancing field being shaped by emerging research and techniques that are enabling increased understanding of brain anatomy and physiology, the pathology of neurological and psychiatric disorders, and viable capabilities and roles of neuromodulation therapies. Since 2012, the DBS Think Tank has been an annual venue for multidisciplinary experts to interactively address challenges, advancements, and opportunities in the field. Convening clinicians, researchers, engineers, ethicists, and industry professionals, the DBS Think Tank has addressed ways of improving clinical outcomes; technological innovations; neurophysiological and imaging-based markers of pathology and response to DBS; emerging indications and targets for DBS; advancements in commercial devices and technologies; and ethical challenges and their potential solutions.

As a vector for disseminating information and with support from the Frontiers Editorial Office, this Research Topic has been developed to focus on topics presented at each year's DBS Think Tank. The proceedings summarizing the annual meetings have also been consistently published as part of this Research Topic, including the recent meetings in 2022 (Wong et al.) and 2023 (Johnson et al.) in the present volume.

In this editorial, we summarize the sixteen studies presented in the fourth volume, which address: (1) improving clinical practice; (2) neuroimaging techniques to optimize DBS targeting; (3) generating deepened insights into the effects of DBS on pathologic symptoms; (4) utility of DBS to treat certain neuropsychiatric disorders; and (5) patient-focused considerations for translational research.

## Improving clinical practice and technology translation

Although DBS is regarded as a primary surgical therapy for Parkinson's disease (PD), access to DBS remains relatively limited. Memon et al. performed a systematic review to evaluate the influence of demographic and socioeconomic factors on patient access to DBS. Their investigation revealed that individuals who were elderly, female, Black, and from low socioeconomic backgrounds and developing countries encountered greater obstacles in accessing DBS for PD. Considering these findings, the authors suggest that strategies engaging all stakeholders to reduce such disparities should be developed and implemented.

DBS is a treatment option for essential tremor (ET); however, the effects of DBS on cognitive outcomes in ET are not well characterized. Al Ali et al. reviewed the existing literature to evaluate whether DBS targeting the ventral intermediate nucleus (VIM) or caudal zona incerta/posterior subthalamic area (cZi/PSA) induced cognitive changes. Their analysis found that measures of verbal cognitive ability declined in some ET patients treated with DBS; however, these changes were not significant, and severe decline was relatively rare. Controlled trials are needed to thoroughly investigate the contributing factors.

Case reports are valuable for sharing challenges associated with DBS and potential clinical indications. Holland et al. reported a patient who received DBS for PD, whereafter a pocket hematoma formed around the implanted pulse generator (IPG), which led to behaviors resembling “Twiddler's syndrome” (i.e., flipping the device within the pocket) and ultimately led to device failure. To prevent this complication, the authors suggested anchoring the IPG to a deep fascial layer or using an antimicrobial pouch.

MacLean et al. reported three patients with childhood-onset dystonia whose axial or orofacial symptoms were refractory to standard pallidal DBS and subsequently underwent DBS targeted to the pedunculopontine nucleus (PPN). All of the patients had clinically significant dystonia improvement but required intensive DBS programming over several months. This case series suggests the PPN may be a potential DBS target for patients from this subpopulation, but larger controlled studies are required for thorough investigation.

## Imaging to optimize stimulation targeting

Imaging is crucial for DBS targeting and understanding the effects of DBS on local neuroanatomy and brain networks. Emerging techniques aim to identify neuroanatomical structures that are not easily delineated in structural MRI. Patriat et al. introduced the novel method of diffusion MRI for anatomical nuclei imaging (DiMANI) to visualize thalamic nuclei in individual patients. DiMANI showed high reproducibility and clinical relevance and thus could refine thalamic DBS targeting approaches.

Computational models of DBS complement imaging by estimating the effects of DBS on local neural structures and networks. Patrick et al. comprehensively reviewed the methods and applications of modeling the volume of tissue activated (VTA) by DBS. The authors compared various VTA methods, parameters, and software platforms available for integrating imaging and computational modeling. Their review can serve as a central resource for clinicians and researchers incorporating imaging and VTA methods.

Imaging and computational models of DBS were employed by Yu et al. to investigate whether DBS targeting should be tailored to ET vs. “ET-plus,” defined as ET and additional neurologic signs, such as impaired gait and dystonic posturing. The authors found no significant differences in the optimal stimulation site or VTA-fiber pathway overlap between groups. However, objective methods to discern ET and ET-plus are needed, and other markers (e.g., electrophysiology) could be valuable to refine DBS methods for distinct patient populations.

## Unraveling the effects of DBS on pathologic symptoms

Major research foci have been on understanding the pathophysiological basis of neurological and neuropsychiatric symptoms and how DBS modulates these symptoms. Munoz et al. studied the effects of subthalamic nucleus (STN) DBS and levodopa medication on eye and limb movements in PD patients using a visually-guided reaching task administered either on/off medications or on/off STN DBS. Levodopa medication worsened visual saccade performance but improved reaching performance, while STN DBS improved both saccade and reaching performance. These findings highlight the importance of evaluating multiple measures when assessing the effects of particular treatments on PD disease state.

Non-motor symptoms of PD are becoming increasingly relevant to DBS therapy. Memon et al. employed EEG-monitored sleep to study the effects of low (60 Hz) and high (≥130 Hz) frequency STN DBS in PD patients with self-reported sleep complaints. Sleep spindle density was significantly higher with high-frequency DBS compared to low-frequency DBS, whereas slow wave activity during non-rapid eye movement (NREM) sleep was increased during low-frequency DBS compared to high-frequency or off DBS. Their findings motivate research toward developing more precise DBS parameters for positive effects on sleep.

## Advancing DBS in neuropsychiatric disorders

Neuropsychiatric symptoms can be a debilitating non-motor component of PD. Muhammad et al. studied the effects of STN DBS on evaluating emotional stimuli in individuals with PD. Subjects participated in emotional picture-viewing tasks while STN local field potentials (LFP) and EEG were recorded. Negative emotionally valent pictures were associated with time-locked, acute STN DBS. With 130 Hz DBS, alpha power decreased in response to negative vs. neutral images irrespective of stimulation. However, with 10 Hz DBS, alpha power was not decreased, but power in the alpha and beta bands were increased. Therefore, low-frequency DBS may synchronize neurophysiological activity, which could potentially guide new DBS paradigms for neuropsychiatric symptoms.

DBS appears promising for treating specific treatment-resistant neuropsychiatric disorders, such as treatment-resistant depression (TRD) and obsessive-compulsive disorder (OCD). Allawala et al. studied the effects of stimulation in the subcallosal cingulate (SCC) or ventral capsule/ventral striatum (VC/VS) on prefrontal neural activity measured with stereo-EEG in two subjects enrolled in an ongoing clinical trial investigating DBS for TRD. DBS in the SCC vs. the VC/VS differentially modulated gamma band activity but in a shared prefrontal circuit. Their findings may provide preliminary evidence of brain networks involved in the therapeutic effects of DBS for TRD.

Studies of DBS in OCD have begun transitioning toward network-guided approaches. Basich-Pease et al. reported a patient who underwent bilateral anterior limb of the internal capsule (ALIC) DBS for the treatment of OCD and TRD. The patient experienced initial modest improvements, but their right lead was not contributing, potentially due to a suboptimal location. Lead repositioning was guided by tractography to target fiber pathways connecting the medial and ventrolateral prefrontal cortices and mediodorsal thalamus. After lead repositioning, the patient's OCD symptoms and subjectively reported quality of life substantially improved. This case highlights the value of tractography in evaluating and guiding DBS electrode targeting and repositioning.

## Patient-focused considerations for translational research

Commercial DBS devices that support chronic recordings have opened new opportunities for studying biomarkers of symptoms and behavior, especially in the naturalistic environment. Feldmann et al. present a patient engagement study for chronic DBS research focused on capturing patients' perspectives on study design, data acquisition, and infrastructure. Involving patients' perspectives in these dimensions of research will be important for defining and implementing strategies to positively affect patient motivation, participation, and compliance.

Identifying biomarkers of symptoms is important for developing patient-focused DBS approaches. Using such biomarkers, adaptive DBS (aDBS) methods can be employed to titrate stimulation in real time according to the patient's symptoms. Wilkins et al. outline key considerations for successfully implementing aDBS, including identifying reliably detected biomarkers and selecting parameters to optimize algorithm performance to meet patients' needs. These considerations are critical to ensure the successful translation of aDBS to clinical applications.

## Conclusions

The studies presented in this volume provide a broad view of the current innovations, challenges, and opportunities of DBS. Iterative advancements in neuroimaging, computational modeling, and electrophysiological techniques have deepened our understanding of the pathophysiology of various neurological and neuropsychiatric disorders and refined targets for DBS to achieve beneficial therapeutic effects. The overarching goal is to translate cutting-edge technology toward developing more efficient and effective DBS approaches. However, challenges remain that impede widespread clinical adoption and employment of DBS, including disparities in access to DBS in underserved populations and in patients with less common disorders who do not have other effective treatment options. Improving the technologies, guidelines, and policies that enable more facile use of DBS will require multidisciplinary collaborations among clinicians, researchers, engineers, ethicists, industry professionals, policymakers, and patients. The DBS Think Tank aims to engage national and international colleagues by providing a venue and resource for current and future collaborations in the field.

## Author contributions

KJ: Writing – review & editing, Writing – original draft. JG: Writing – review & editing, Writing – original draft. AR-Z: Writing – review & editing, Writing – original draft. MS: Writing – review & editing, Writing – original draft. SM: Writing – review & editing, Writing – original draft. MO: Writing – review & editing, Writing – original draft. JW: Writing – review & editing, Writing – original draft.

